# Prevalence of financial toxicity and construction of a risk prediction model for lung cancer patients at 6 months after discharge

**DOI:** 10.3389/fmed.2026.1895554

**Published:** 2026-09-03

**Authors:** Ningning Liu, Xiaoyan Wang, Xiaofeng Pan, Caifang Tang, Ximiao Li, Yiting Zhang, Yaru Zhao

**Affiliations:** Shanghai Songjiang District Sijing Hospital, Shanghai, China

**Keywords:** financial toxicity, lung cancer, patient-reported outcome, prospective cohort study, risk prediction model

## Abstract

**Objective:**

To investigate the prevalence of 6-month post-discharge financial toxicity in lung cancer patients, identify its independent predictors, and establish a valid risk prediction model integrating socioeconomic, clinical, and patient-reported outcome variables as baseline predictors, so as to support early financial toxicity screening, targeted financial counseling and standardized nurse-led stratified transitional nursing interventions.

**Methods:**

A single-center prospective cohort study enrolled 682 hospitalized lung cancer patients. Baseline demographic, socioeconomic, clinical, and patient-reported data (resilience, social support, fear of progression, symptom burden, self-efficacy) were collected. financial toxicity was assessed via the COST-PROM at 6 months post-discharge (score ≤ 25 defined as financial toxicity). Multivariable logistic regression was used to build the model, with ROC curve, calibration test, calibration slope, calibration intercept, optimism, shrinkage factor, Decision Curve Analysis, and 500-time Bootstrap resampling internal validation.

**Results:**

The 6-month post-discharge financial toxicity incidence was 76.2%. Annual household income, long-term residence, immunotherapy, self-efficacy, resilience, and fear of progression were independent predictors (*P* < 0.05). The model AUC was 0.901 (Bootstrap-corrected: 0.894), with an optimal cut-off of 0.757 (sensitivity: 0.800, specificity: 0.852). The Hosmer-Lemeshow test confirmed good calibration, and risk subgroups showed increasing financial toxicity incidence, demonstrating favorable stratification ability.

**Conclusion:**

Lung cancer patients/survivors suffer from substantial financial toxicity within 6 months after hospital discharge, with household economic status, residential location, treatment modality, and diverse psychosocial coping factors serving as critical joint predictors of financial toxicity risk. The newly constructed prediction model underwent rigorous 500-time Bootstrap internal validation and demonstrated favorable discrimination, calibration, stability and overall predictive efficacy. This model enables clinicians and nurses to identify patients at high risk of financial toxicity before discharge, conduct early financial toxicity assessment, provide preliminary guidance on healthcare costs and insurance information, and coordinate tiered transitional care interventions through multidisciplinary collaboration to address cancer-related financial distress. Nevertheless, external validation remains essential before formal clinical implementation of the model.

## 1 Introduction

Lung cancer is one of the malignant tumors with the heaviest disease burden worldwide (1). With advances in comprehensive treatment modalities, including surgery, radiotherapy, chemotherapy, targeted therapy, and immunotherapy, the survival of patients with lung cancer has been prolonged. However, long-term, continuous, and multimodal treatment also imposes a sustained economic burden on patients and their families (2). In addition to direct medical costs, non-medical expenses such as examinations, transportation, caregiving, and income loss can further aggravate the economic burden on patients (3). For lung cancer patients requiring long-term treatment and follow-up management, the economic burden is not merely a matter of payment; it may also affect treatment adherence, psychological status, symptom experience, quality of life, and subsequent disease management processes (4).

Financial toxicity is an important concept in recent oncology supportive care and patient-reported outcome research. It is commonly used to describe the objective economic burden and subjective financial distress caused by a combination of medical costs, non-medical expenses, and income loss during the cancer diagnosis and treatment process (5). Unlike a mere increase in medical costs, financial toxicity places greater emphasis on the patient’s comprehensive experience of payment pressure, diminished financial capacity, work impairment, and compromised quality of life over the course of treatment (6). Previous studies have shown that financial toxicity may be associated with treatment delays, reduced medication use, decreased follow-up adherence, increased psychological distress, and diminished health-related quality of life (7). Therefore, timely identification of patients at high risk for financial toxicity is of great significance for implementing cost counseling, health insurance policy explanation, social support linkage, psychological care, and continuous care. The COST-PROM is currently one of the widely used standardized patient-reported outcome instruments in research on financial toxicity among cancer patients. It can be used to assess patients’ subjective perceptions of financial stress, cost burden, work impact, and financial distress (8).

The occurrence of financial toxicity in patients with lung cancer is characterized by a multifactorial nature. On the one hand, clinical factors such as disease stage, presence of metastasis, treatment modality, number of hospitalizations, and symptom burden may increase patients’ treatment needs and care costs. On the other hand, socioeconomic factors including income level, source of medical expenses, insurance type, employment status, family structure, and place of residence can also affect patients’ ability to afford medical expenditures (9). Moreover, patient-reported outcome variables such as psychological resilience, social support, fear of disease progression, symptom burden, and self-efficacy may influence patients’ perception of economic pressure, coping styles, and capacity to utilize resources (10). While prospective studies on FT in lung cancer exist [e.g., (11)], most were conducted over 4 years ago and focused on general oncology populations or short-term follow-up, with limited integration of multi-dimensional PROs. Existing studies have mostly focused on the current status of financial toxicity in patients with lung cancer and the analysis of associated factors. Existing studies have mostly focused on the current status of financial toxicity in patients with lung cancer and the analysis of associated factors. Although several risk prediction models for financial toxicity have been developed (12, 13), these models predominantly rely on demographic and clinical indicators and have not incorporated multi-dimensional patient-reported psychosocial factors such as resilience, self-efficacy, and fear of progression. Moreover, most existing tools were designed to assess in-hospital or short-term financial burden rather than the cumulative economic pressure during the mid-term post-discharge follow-up period. Given that lung cancer treatment is characterized by long-term, continuous, and multimodal regimens, the dynamic interplay between psychosocial resources and financial distress during the follow-up phase remains underexplored. Therefore, we developed a new prediction model specifically for 6-month post-discharge financial toxicity by integrating socioeconomic, clinical, and patient-reported outcome variables, aiming to fill this gap and provide a practical tool for early risk stratification and targeted nursing interventions during transitional care.

From a nursing practice perspective, financial toxicity in patients with lung cancer should not be identified only at the time of cost settlement or when patients actively seek help. Nurses have frequent contact with patients during hospitalization and can identify early risk signals related to treatment costs, household ability to pay, work interruption, psychological stress, and symptom burden (14).

Financial toxicity is characterized by a staged and cumulative nature, and its risk may change continuously over the course of diagnosis, inpatient treatment, post-discharge follow-up visits, adjuvant therapy, and systemic therapy (15). For lung cancer, however, the trajectory of financial toxicity may differ, as evidence suggests that its severity tends to improve during the follow-up period. Compared with assessment at the time of discharge, the sixth month after discharge better reflects the ongoing treatment costs, costs of follow-up examinations, non-medical expenses, and income recovery after patients enter the follow-up maintenance period, and it also aligns more closely with the time window for transitional care and mid-term intervention (16, 17). Evaluation immediately after discharge primarily captures one-off inpatient expenses and may not adequately reflect the cumulative costs of maintenance therapy and long-term care. Short-term follow-up of 1–3 months may be insufficient to capture the gradual accumulation of economic stress, whereas follow-up beyond 12 months is susceptible to numerous confounding changes in patients’ household financial circumstances. The 6-month time point represents a balance between stable baseline data derived from hospitalization and the full presentation of cumulative financial burden during the follow-up period. Therefore, using the occurrence of financial toxicity at the sixth month after discharge as the predictive outcome can help evaluate the predictive value of information available during hospitalization for patients’ mid-term economic burden, and provide a basis for risk stratification and nursing resource allocation during the follow-up phase.

Based on this, the present study recruited inpatients with lung cancer as participants. Demographic data, socioeconomic data, clinical data, and patient-reported outcome scale scores were collected during hospitalization, and the occurrence of financial toxicity was assessed using the COST-PROM at the sixth month after discharge. This study aimed to analyze the predictors of financial toxicity in patients with lung cancer at 6 months after discharge and to construct a risk prediction model, so as to provide a reference for early identification of financial toxicity, development of discharge plans, prioritization of follow-up visits, and stratified nursing management in this population.

## 2 Materials and methods

### 2.1 Study design

This was a single-center prospective cohort study. Based on demographic, socioeconomic, clinical data and patient-reported outcomes collected during hospitalization of lung cancer patients, this study aimed to identify predictors of financial toxicity at 6 months after discharge and develop a risk prediction model for 6-month post-discharge financial toxicity in lung cancer patients. The design and reporting of this study complied with the Strengthening the Reporting of Observational Studies in Epidemiology (STROBE) and the Transparent Reporting of a multivariable prediction model for Individual Prognosis Or Diagnosis (TRIPOD) guidelines (18, 19).

Data collected during hospitalization were used as baseline predictive variables, and the occurrence of financial toxicity at 6 months after discharge was set as the primary outcome. Financial toxicity was assessed using the Comprehensive Score for Financial Toxicity based on Patient-Reported Outcome Measures (COST-PROM). A total COST-PROM score ≤ 25 was defined as the presence of financial toxicity, while a score > 25 indicated no financial toxicity (10). This model was developed to predict the risk of financial toxicity at 6 months post-discharge, rather than to estimate causal relationships between predictive variables and financial toxicity.

### 2.2 Study setting and participants

Participants were recruited from April 2025 to November 2025 at a general hospital in Shanghai, China. During the study period, all hospitalized patients with lung cancer who met the predefined inclusion and exclusion criteria were consecutively screened and invited to participate. Eligible patients who agreed to participate were enrolled and followed up until May 2026 to complete the 6-month post-discharge assessment. Baseline demographic, socioeconomic, clinical, and patient-reported outcome data were collected during hospitalization, and financial toxicity was assessed at 6 months after discharge.

The inclusion criteria were as follows:

(1) Aged 18 years or older;
(2) Diagnosed with lung cancer via pathological or cytological examination;
(3) Possessing complete clinical and medical records;
(4) Being fully conscious with normal communication, comprehension and questionnaire response abilities;
(5) Being willing to complete the 6-month follow-up after hospital discharge;
(6) Voluntarily participating in this study and signing the informed consent form.

Exclusion criteria were as follows:

(1) Patients with a history of mental illness, cognitive impairment, intellectual disability or other severe psychological disorders who were unable to finish the questionnaires;
(2) Patients complicated with severe dysfunction of vital organs such as the heart, brain and kidney and unable to cooperate with the investigation;
(3) Patients with severe physical dysfunction and complete dependence in daily life who could not complete patient-reported outcome assessments;
(4) Patients with missing data on key baseline predictive variables;
(5) Patients with missing data regarding the outcome of financial toxicity at 6 months after discharge;
(6) Patients who voluntarily withdrew from the study during the investigation.

### 2.3 Sample size calculation

This study aimed to develop a risk prediction model for financial toxicity among lung cancer patients at 6 months after discharge. The sample size was calculated considering both the estimation of the incidence of financial toxicity and the stability of the multivariate logistic regression model.

Based on previous studies and preliminary survey results, the incidence of financial toxicity among cancer patients varies across different research settings. To ensure a conservative sample size estimation, the formula for population proportion estimation was adopted. With α = 0.05, the two-tailed test corresponding to Uα/2 = 1.96 and an allowable error δ = 0.05, the required sample size was approximately 384 cases when calculated based on the maximum variability π = 0.50. Considering a loss to follow-up or invalid data rate of 10–15%, at least 427–452 participants should be enrolled in this study.

Additionally, a multivariate logistic regression prediction model was constructed in this study, so the rationality of sample size was further evaluated based on the ratio of outcome events to degrees of freedom in the model. To reduce the risk of overfitting and ensure stable regression estimation, a minimum of 10 outcome events were required for each degree of freedom in the prediction model. According to the candidate predictive variables planned for inclusion and the principle of model parsimony, the number of degrees of freedom in the final model was controlled within 10–12, which required at least 100–120 outcome events. Combining the estimation of population proportion, the stability of model construction, and a 10–15% rate of loss to follow-up and invalid data, the target sample size of this study was set to no less than 500 cases.

Ultimately, a total of 682 lung cancer patients completed the 6-month post-discharge follow-up and were included in the analysis, among whom 520 developed financial toxicity. The final multivariate logistic regression model contained 8 degrees of freedom, with an event-to-parameter ratio of 65.0, which met the basic requirements for developing a prediction model. This sample size scheme strictly complies with contemporary TRIPOD guidance for prediction model construction. Dual standards of population prevalence estimation and event-per-variable (EPV) principle were integrated to calculate target sample size, which is the mainstream standardized method recommended in recent predictive epidemiology literatures. The final EPV of 65.0 greatly surpasses the minimum threshold of 10 EPV advocated by modern methodological research, effectively reducing the risk of model overfitting and ensuring stable regression coefficient estimation and credible internal validation results.

### 2.4 Outcome variables and candidate variables

#### 2.4.1 Outcome variables

The primary outcome variable was the occurrence of financial toxicity in lung cancer patients at 6 months after discharge. The COST-PROM was used to assess the level of financial toxicity at this time point.

The COST-PROM consists of 11 items to evaluate patients’ perceptions of financial stress, economic burden, work impacts and finance-related distress over the preceding 7 days. All items are rated on a 5-point Likert scale, with total scores ranging from 0 to 44. Lower scores indicate more severe financial toxicity. Based on the cut-off value validated and widely adopted in previous studies among Chinese cancer populations, financial toxicity was defined as a total COST-PROM score ≤ 25 at 6 months post-discharge, while a score > 25 indicated the absence of financial toxicity (10). A binary outcome variable was established accordingly.

#### 2.4.2 Potential predictive variables

Candidate predictive variables were selected based on literature review (12, 13), clinical relevance, accessibility in nursing assessment and the timeline of variable measurement. The selection of patient-reported outcome variables—resilience, social support, fear of progression, symptom burden, and self-efficacy—was guided by prior evidence linking them to financial toxicity (10, 20–22). Specifically, self-efficacy and social support mediate the effects of socioeconomic status on financial outcomes (10); resilience buffers financial distress (22); fear of progression exacerbates perceived burden (21); and symptom burden impairs work capacity and increases out-of-pocket costs (20). All candidate variables were collected during hospitalization or prior to discharge, earlier than the assessment of financial toxicity at 6 months after discharge. These variables were divided into demographic and socioeconomic variables, lung cancer-related clinical variables, and patient-reported outcome variables.

##### 2.4.2.1 Demographic and socioeconomic variables

These included sex, age, permanent residence, educational level, marital status, number of children, living status, employment status before diagnosis, current employment status, occupation type, annual household income, source of medical expenses, type of medical insurance, and travel time to the medical institution. Age was recorded as a continuous variable, and other variables were categorized according to predefined criteria. All demographic and socioeconomic data were collected via patient self-report. Annual household income was classified into four levels: < 20,000 CNY, 20,000–50,000 CNY, 50,000–100,000 CNY, and ≥ 100,000 CNY, with the lowest category ( < 20,000 CNY) designated as the reference group in subsequent modeling, because this allows the odds ratios for higher income levels to be directly interpreted as protective effects relative to the group at greatest financial risk.

##### 2.4.2.2 Lung cancer-related clinical variables

These comprised time since diagnosis, clinical stage, presence of metastasis, pathological type, comorbidities, history of surgery, chemotherapy, radiotherapy, targeted therapy and immunotherapy, as well as the number of hospital admissions within the past year. Clinical data were extracted and verified by researchers following unified variable definitions from the electronic medical record system.

##### 2.4.2.3 Patient-reported outcome variables

Resilience, social support, fear of disease progression, symptom burden and self-efficacy were included in this section, which were measured using the Connor-Davidson Resilience Scale, Social Support Rating Scale, Short Form of Fear of Progression Questionnaire, M.D. Anderson Symptom Inventory and General Self-Efficacy Scale, respectively. All scale assessments were completed during hospitalization or before discharge to reflect patients’ baseline psychosocial status and symptom burden.

Resilience was measured using the Connor-Davidson Resilience Scale (CD-RISC) (23). This scale contains 25 items rated on a 5-point Likert scale, with total scores ranging from 0 to 100. Higher scores indicate greater resilience.

Social support was assessed with the Social Support Rating Scale (SSRS) (24). It consists of 10 items covering three dimensions: objective support, subjective support and utilization of social support. A higher total score represents a higher level of social support.

Fear of disease progression was evaluated by the Fear of Progression Questionnaire-Short Form (FoP-Q-SF) (25). This 12-item scale includes two dimensions of social-family issues and physical health. All items are scored on a 5-point Likert scale, with total scores from 12 to 60. Higher scores reflect stronger fear of disease progression.

Symptom burden was measured via the M. D. Anderson Symptom Inventory (MDASI) (26). The inventory assesses symptom severity and interference with daily life, with each item scored from 0 to 10. The total item score was adopted for analysis in this study, and a higher score suggests heavier symptom burden.

General self-efficacy was tested using the General Self-Efficacy Scale (GSES) (27). This scale has 10 items with a 4-point Likert scoring system. Total scores range from 10 to 40, and higher scores correspond to stronger self-efficacy.

### 2.5 Data collection and quality control

Prior to formal data collection, the research team developed standardized data collection forms, variable definition sheets and follow-up records. All investigators received unified training covering research objectives, inclusion and exclusion criteria, variable definitions, questionnaire specifications, standardized instructions, extraction of electronic medical record data, follow-up procedures, data entry rules and privacy protection protocols. A pilot survey was conducted among 15 eligible lung cancer patients to evaluate item clarity, completion time, data collection procedures and follow-up feasibility. Relevant instructions and workflows were revised according to the pilot results.

Baseline data were collected during patients’ hospitalization. Demographic and socioeconomic information including gender, age, permanent residence, educational level, marital status, number of children, living arrangement, pre-diagnosis and current employment status, occupation, annual household income, source of medical expenses, type of medical insurance and travel time to the hospital were obtained through patient self-report. Clinical data such as time since diagnosis, clinical stage, metastasis status, pathological type, comorbidities, treatment regimens and hospital admission frequency in the past year were extracted from the electronic medical record system. Trained investigators administered face-to-face questionnaires with standardized instructions to collect patient-reported outcomes, including scores of resilience, social support, fear of disease progression, symptom burden and self-efficacy. All baseline predictive variables were collected prior to the assessment of financial toxicity at 6 months after discharge.

Follow-up assessments for financial toxicity were performed at 6 months after discharge. Assessments were conducted via on-site interviews during outpatient visits, telephone interviews or online questionnaires. Follow-up staff explained the completion requirements of the COST-PROM to patients using unified instructions and recorded the total COST-PROM score at the 6-month follow-up. For patients who failed to complete follow-up on schedule, researchers made at least three attempts to establish contact within the predefined follow-up window. Cases with no valid outcome data after repeated attempts were recorded as loss to follow-up and excluded from the analysis.

Data quality was ensured through standardized investigator training, independent double-entry of questionnaire data with consistency verification, and cross-checking of clinical variables extracted from electronic medical records by two researchers. Data with logical contradictions or outliers were verified against original sources, and unverifiable data were handled according to pre-established rules. Complete primary outcome data were required for final analysis.

### 2.6 Bias control

To reduce selection bias, participants were enrolled consecutively. All eligible lung cancer inpatients who met the inclusion and exclusion criteria were recruited during the study period, and participants were screened strictly following uniform criteria. Baseline data were collected during hospitalization to minimize selection bias caused by changes in disease status, treatment regimens or differential follow-up accessibility after discharge. Reasons for loss to follow-up were documented for patients who failed to complete the 6-month post-discharge assessment. Only participants with complete primary outcome data were included in the final model analysis.

To mitigate information bias and measurement bias, standardized training and unified instructions were used. Demographic and socioeconomic data were collected via standardized forms. Clinical data were extracted from electronic medical records and verified according to predefined criteria; patient-reported outcomes were assessed using validated standardized scales.

To reduce follow-up bias, at enrollment, at least one valid contact method was recorded for each participant. The 6-month post-discharge follow-up was conducted via outpatient visits, telephone calls or online questionnaires. Multiple contact attempts were made within the predefined follow-up window for participants who missed scheduled assessments, and reasons for loss to follow-up were documented. Complete case data were used for the final analysis, and information on participant screening, exclusion and loss to follow-up was summarized in accordance with the STROBE and TRIPOD guidelines.

To control confounding and model bias, candidate predictive variables were selected based on published evidence, clinical relevance, accessibility in nursing assessment and measurement timeline. All candidate variables were collected prior to the assessment of 6-month post-discharge financial toxicity. Continuous variables were incorporated into the model in their original form to avoid information loss resulting from unnecessary categorization. Polytomous variables were converted into dummy variables before modeling, with clinically meaningful reference groups assigned. The number of candidate variables and degrees of freedom in the final model were strictly controlled. The event-to-parameter ratio was calculated to evaluate model stability and reduce the risk of overfitting.

Bootstrap resampling was applied for internal validation to further mitigate overfitting and optimism bias of the prediction model, so as to assess the stability of model discrimination and calibration. The Bootstrap-corrected AUC, calibration curve, Hosmer-Lemeshow goodness-of-fit test, Brier score and key classification metrics were reported to comprehensively evaluate the predictive performance of the model.

To address potential selection bias arising from the exclusion of participants with missing primary outcome data, we conducted a systematic assessment of missing data patterns. The overall missing proportion for all candidate baseline predictors was below 2%, and missingness was not associated with the 6-month financial toxicity outcome, supporting a missing-completely-at-random mechanism. In addition to the pre-specified complete-case analysis, we performed a sensitivity analysis using multiple imputation for baseline predictors; the regression coefficients and model performance metrics derived from imputed datasets were highly consistent with those from the complete-case analysis, indicating that our missing data handling strategy was robust and unlikely to introduce substantial bias.

### 2.7 Statistical analysis and model development

All statistical analyses were performed using IBM SPSS Statistics 27.0 and R software. Participants were divided into the financial toxicity group and non-financial toxicity group according to the 6-month post-discharge COST-PROM total score.

Descriptive analyses were conducted for baseline demographic, socioeconomic, clinical data and scores of patient-reported outcome scales. Normality tests were performed for continuous variables. Normally distributed data were presented as mean ± standard deviation and compared using the independent-samples *t*-test; non-normally distributed data were described as median and interquartile range and analyzed via the Mann-Whitney U test. Categorical variables were summarized as frequencies and percentages, and compared using the Chi-square test or Fisher’s exact test.

The presence of financial toxicity at 6 months after discharge was set as the dependent variable, which was coded as 0 for no financial toxicity and 1 for financial toxicity. Candidate predictive variables were predefined based on literature evidence (10, 12, 13, 20–22), clinical relevance, accessibility in nursing assessment and measurement sequence. Univariate analysis was used to explore between-group differences and provide references for model development, rather than serving as the sole screening criterion. The final multivariable model retained only predictors with sufficient event counts and stable estimates to ensure model reliability. All candidate variables were derived from baseline data collected during hospitalization or before discharge, prior to the assessment of the primary outcome. Polytomous variables were converted into dummy variables before modeling, with categories of clear clinical significance or large sample size set as reference groups. Continuous variables were included in the model in their original form in principle. Given that small unit changes in scale total scores may lead to unintuitive interpretation of odds ratios (ORs), ORs were calculated per 10-point increment for the General Self-Efficacy Scale and Fear of Progression Questionnaire-Short Form in the multivariate logistic regression model.

A multivariate logistic regression model was established to predict the risk of financial toxicity among lung cancer patients at 6 months after discharge. Variable selection took into account clinical implications, statistical results, measurement timeline and model parsimony. The event-to-parameter ratio was calculated to assess model stability and reduce overfitting. Results of the multivariate logistic regression were presented as regression coefficient β, standard error, odds ratio (OR) and corresponding 95% confidence interval (CI). An OR > 1 indicated a higher predicted probability of financial toxicity, while an OR < 1 indicated a lower probability. These findings were only used for risk prediction rather than causal inference. All statistical tests were two-tailed, and a *P* < 0.05 was considered statistically significant. For variable selection, all candidate predictors with *P* < 0.05 in univariate analyses together with clinically meaningful variables without statistical significance were retained for multivariable regression. Backward elimination was adopted with removal threshold *P* > 0.10; clinical relevance and model parsimony were prioritized over pure statistical significance during elimination to avoid excluding clinically critical risk factors. No automated fully stepwise selection without clinical judgment was used.

Model discrimination was evaluated using the receiver operating characteristic (ROC) curve and the area under the curve (AUC), with the AUC and its 95% CI reported. The optimal cut-off value was determined by the Youden index, and sensitivity, specificity, positive predictive value and negative predictive value were calculated to assess classification performance. Model calibration was assessed via calibration curve, Hosmer-Lemeshow goodness-of-fit test and Brier score, calibration slope and calibration intercept. To evaluate and correct for overfitting, we performed 500 bootstrap resamples to calculate the optimism and shrinkage factor. Decision Curve Analysis (DCA) was conducted to evaluate the clinical net benefit of the model across a range of threshold probabilities. All these analyses were performed using R software (version 4.5.3). Internal validation was performed using 500 Bootstrap resamples, and the Bootstrap-corrected AUC was reported to evaluate model stability and overfitting risk. Prior to multivariate regression fitting, multicollinearity among all candidate predictors was quantified via variance inflation factor (VIF). Variables with VIF > 10 would be eliminated or combined to eliminate collinearity bias; all predictors retained in the final model had VIF < 3, indicating negligible multicollinearity. The linearity assumption between continuous predictors and logit of outcome was verified via Box-Tidwell test: interaction terms of each continuous variable and its natural logarithm were introduced into preliminary regression, and all interaction terms showed *P* > 0.05, confirming linear relationships and satisfying the core assumption of logistic regression.

To further assess risk stratification capacity, participants were divided into deciles according to predicted probabilities. The mean predicted risk and actual incidence of financial toxicity were compared across deciles. Participants were also classified into low-, medium- and high-risk groups based on tertiles of predicted probabilities, and the actual incidence of financial toxicity was compared among the three groups.

For missing data, original questionnaires, follow-up records and electronic medical records were reviewed first. Cases with missing primary outcome data were excluded from the final analysis. Missing baseline predictive variables were handled according to missing rate and missing pattern. Complete case analysis was adopted when the missing rate of key variables was low. If the missing rate was high, missing information was clearly reported, and its potential impact on model results was evaluated. We further supplemented comprehensive missing data assessment: the missing proportion and missing mechanism (missing completely at random / missing at random) of each baseline predictor were counted. All variables entering the final prediction model had an overall missing rate below 2%, without missing values correlated with the 6-month financial toxicity outcome. Patients lacking primary COST-PROM outcome data were excluded via complete case analysis as prespecified in the research protocol. Sensitivity analysis based on multiple imputation was additionally conducted; regression coefficients from imputed datasets were highly consistent with primary complete-case results, proving our missing data handling strategy robust.

### 2.8 Ethical considerations

This study was approved by the Ethics Committee of Sijing Hospital, Songjiang District, Shanghai (Ethics Approval No. SJYY2024-KYLW-ZYR). All participants signed written informed consent after being fully informed of the research objectives, survey contents, potential risks and data confidentiality rules. The principles of voluntary participation, informed consent, privacy protection and data confidentiality were strictly implemented throughout the study. All collected data were only used for analytical purposes of this research.

## 3 Results

### 3.1 Participant enrollment and incidence of financial toxicity at 6 months after discharge

A total of 682 patients completed the 6-month post-discharge follow-up assessment and were included in the final analysis. All included participants had complete COST-PROM outcome data and financial toxicity grouping information. No missing data were observed for the variables included in the final multivariable prediction model after data verification. Among them, 520 patients had a COST-PROM total score ≤ 25 at the sixth month after discharge, yielding an incidence of financial toxicity at 6 months after discharge of 76.2%; 162 patients had a COST-PROM total score > 25, accounting for 23.8%. The median COST-PROM total score at the sixth month after discharge for the entire sample was 18 (interquartile range, 13–25). Detailed information on the incidence of financial toxicity is presented in Table 1.

**TABLE 1:** Incidence of financial toxicity among study participants.

Indicator	n	Proportion (%)	Description
Total sample size	682	–	–
Absence of financial toxicity	162	23.8	COST-PROM > 25
Presence of financial toxicity	520	76.2	COST-PROM ≤ 25
COST-PROM total score	18 (13, 25)	–	Median (P25, P75)

### 3.2 Univariate analysis of factors associated with financial toxicity at 6 months after discharge

Univariate analysis was performed with the occurrence of financial toxicity at the sixth month after discharge as the grouping variable. The results showed that age, long-term place of residence, educational level, marital status, number of children, employment status before diagnosis, current employment status, occupation, annual household income, source of medical expenses, insurance type, travel time to the medical institution, time since diagnosis, clinical stage, presence of metastasis, surgery, radiotherapy, immunotherapy, number of hospitalizations in the past year, as well as the scores of the CD-RISC, SSRS, FoP-Q-SF, MDASI, and GSES differed significantly between the two groups (all *P* < 0.05). Detailed results of the univariate analyses of demographic and socioeconomic characteristics, clinical characteristics, and continuous variables and scale scores are presented in Tables 2–4, respectively.

**TABLE 2:** Univariate analysis of demographic and socioeconomic characteristics.

Variable	Absence of financial toxicity (*n* = 162)	Presence of financial toxicity (*n* = 520)	χ^2^ value	*P*-value
Sex, n (%)			0.950	0.330
Male	109 (67.3)	328 (63.1)		
Female	53 (32.7)	192 (36.9)		
Long-term place of residence, n (%)			89.704	< 0.001
Urban	141 (87.0)	232 (44.6)		
Rural	21 (13.0)	288 (55.4)		
Educational level, n (%)			84.172	< 0.001
Junior high school or below	39 (24.1)	322 (61.9)		
Senior high school/technical secondary school/junior college	83 (51.2)	164 (31.5)		
Bachelor’s degree or above	40 (24.7)	34 (6.5)		
Marital status, n (%)			15.406	< 0.001
Married	147 (90.7)	476 (91.5)		
Unmarried	9 (5.6)	5 (1.0)		
Divorced or widowed	6 (3.7)	39 (7.5)		
Number of children, n (%)			75.498	< 0.001
0	12 (7.4)	12 (2.3)		
1	117 (72.2)	202 (38.8)		
2	29 (17.9)	257 (49.4)		
≥ 3	4 (2.5)	49 (9.4)		
Living alone, n (%)			0.099	0.752
Yes	15 (9.3)	44 (8.5)		
No	147 (90.7)	476 (91.5)		
Employment status before diagnosis, n (%)				< 0.001*
Employed	76 (46.9)	206 (39.6)		
Retired	78 (48.1)	178 (34.2)		
Unemployed	1 (0.6)	15 (2.9)		
Other	7 (4.3)	121 (23.3)		
Current employment status, n (%)			30.226	< 0.001
On leave due to cancer	59 (36.4)	119 (22.9)		
Unemployed due to cancer	5 (3.1)	75 (14.4)		
Retired due to cancer	18 (11.1)	27 (5.2)		
Other	80 (49.4)	299 (57.5)		
Occupation, n (%)			86.144	< 0.001
Public institution/civil servant	57 (35.2)	46 (8.8)		
Enterprise/worker	62 (38.3)	218 (41.9)		
Company employee	20 (12.3)	47 (9.0)		
Self-employed	9 (5.6)	41 (7.9)		
Freelancer	5 (3.1)	33 (6.3)		
Other	9 (5.6)	135 (26.0)		
Annual household income, n (%)			147.253	< 0.001
<20,000 CNY	4 (2.5)	167 (32.1)		
20,000–50,000 CNY	39 (24.1)	208 (40.0)		
50,000–100,000 CNY	71 (43.8)	123 (23.7)		
≥ 100,000 CNY	48 (29.6)	22 (4.2)		
Source of medical expenses, n (%)				< 0.001*
Employee medical insurance	129 (79.6)	213 (41.0)		
Resident medical insurance	21 (13.0)	53 (10.2)		
New Rural Cooperative Medical Insurance	12 (7.4)	236 (45.4)		
Self-paid	0 (0.0)	18 (3.5)		
Insurance type, n (%)				< 0.001*
Social insurance	90 (55.6)	290 (55.8)		
Commercial insurance	2 (1.2)	17 (3.3)		
Both	47 (29.0)	53 (10.2)		
No insurance	23 (14.2)	160 (30.8)		
Smoking history, n (%)			0.094	0.760
Yes	100 (61.7)	314 (60.4)		
No	62 (38.3)	206 (39.6)		
Family history, n (%)			0.046	0.830
Yes	18 (11.1)	61 (11.7)		
No	144 (88.9)	459 (88.3)		
Travel time to the medical institution, n (%)			68.640	< 0.001
<1 h	24 (14.8)	19 (3.7)		
1–2 h	79 (48.8)	137 (26.3)		
2–3 h	37 (22.8)	182 (35.0)		
≥ 3 h	22 (13.6)	182 (35.0)		
Time since diagnosis, n (%)			28.838	< 0.001
<1 month	14 (8.6)	26 (5.0)		
1–6 months	95 (58.6)	199 (38.3)		
6–12 months	24 (14.8)	145 (27.9)		
> 12 months	29 (17.9)	150 (28.8)		

*Fisher’s exact test.

**TABLE 3:** Univariate analysis of clinical characteristics,

Variable	Absence of financial toxicity (*n* = 162)	Presence of financial toxicity (*n* = 520)	χ^2^ value	*P-*value
Clinical stage, n (%)				0.040*
Stage I	4 (2.5)	13 (2.5)		
Stage II	9 (5.6)	20 (3.8)		
Stage III	77 (47.5)	189 (36.3)		
Stage IV	72 (44.4)	298 (57.3)		
Presence of metastasis, n (%)			7.220	0.007
Yes	59 (36.4)	252 (48.5)		
No	103 (63.6)	268 (51.5)		
Pathological type, n (%)				0.102*
Adenocarcinoma	85 (52.5)	231 (44.4)		
Squamous cell carcinoma	33 (20.4)	98 (18.8)		
Small cell carcinoma	42 (25.9)	180 (34.6)		
Other	2 (1.2)	11 (2.1)		
Comorbidity, n (%)			0.072	0.788
Yes	57 (35.2)	177 (34.0)		
No	105 (64.8)	343 (66.0)		
Chemotherapy, n (%)			0.198	0.656
Yes	155 (95.7)	493 (94.8)		
No	7 (4.3)	27 (5.2)		
Surgery, n (%)			12.183	< 0.001
Yes	74 (45.7)	160 (30.8)		
No	88 (54.3)	360 (69.2)		
Radiotherapy, n (%)			5.489	0.019
Yes	19 (11.7)	103 (19.8)		
No	143 (88.3)	417 (80.2)		
Targeted therapy, n (%)			0.329	0.566
Yes	36 (22.2)	127 (24.4)		
No	126 (77.8)	393 (75.6)		
Immunotherapy, n (%)			10.112	0.001
Yes	34 (21.0)	178 (34.2)		
No	128 (79.0)	342 (65.8)		
Number of hospitalizations in the past year, n (%)			25.137	< 0.001
1–3	36 (22.2)	91 (17.5)		
4–5	77 (47.5)	158 (30.4)		
6–9	38 (23.5)	194 (37.3)		
≥ 10	11 (6.8)	77 (14.8)		

*Fisher’s exact test.

**TABLE 4:** Comparison of continuous variables and scale scores between groups.

Variable	Absence of financial toxicity (*n* = 162)	Presence of financial toxicity (*n* = 520)	*Z*-value	*P*-value
Age, years	59 (49, 67)	64 (56, 69)	−3.765	< 0.001
CD-RISC score	56 (50, 60)	44 (37, 50)	11.518	< 0.001
SSRS score	48 (42, 51)	41 (38, 45)	8.491	< 0.001
FoP-Q-SF score	28 (26, 31)	31 (28, 34)	−7.461	< 0.001
MDASI score	58 (46, 79)	77 (56, 97)	−6.518	< 0.001
GSES score	27 (24, 30)	20 (17, 25)	12.259	< 0.001

Data are presented as median (P25, P75). CD-RISC, Connor-Davidson Resilience Scale; SSRS, Social Support Rating Scale; FoP-Q-SF, Fear of Progression Questionnaire-Short Form; MDASI, MD Anderson Symptom Inventory; GSES, General Self-Efficacy Scale.

### 3.3 Multivariable logistic regression analysis of predictors of financial toxicity at 6 months after discharge

**FIGURE 1:**
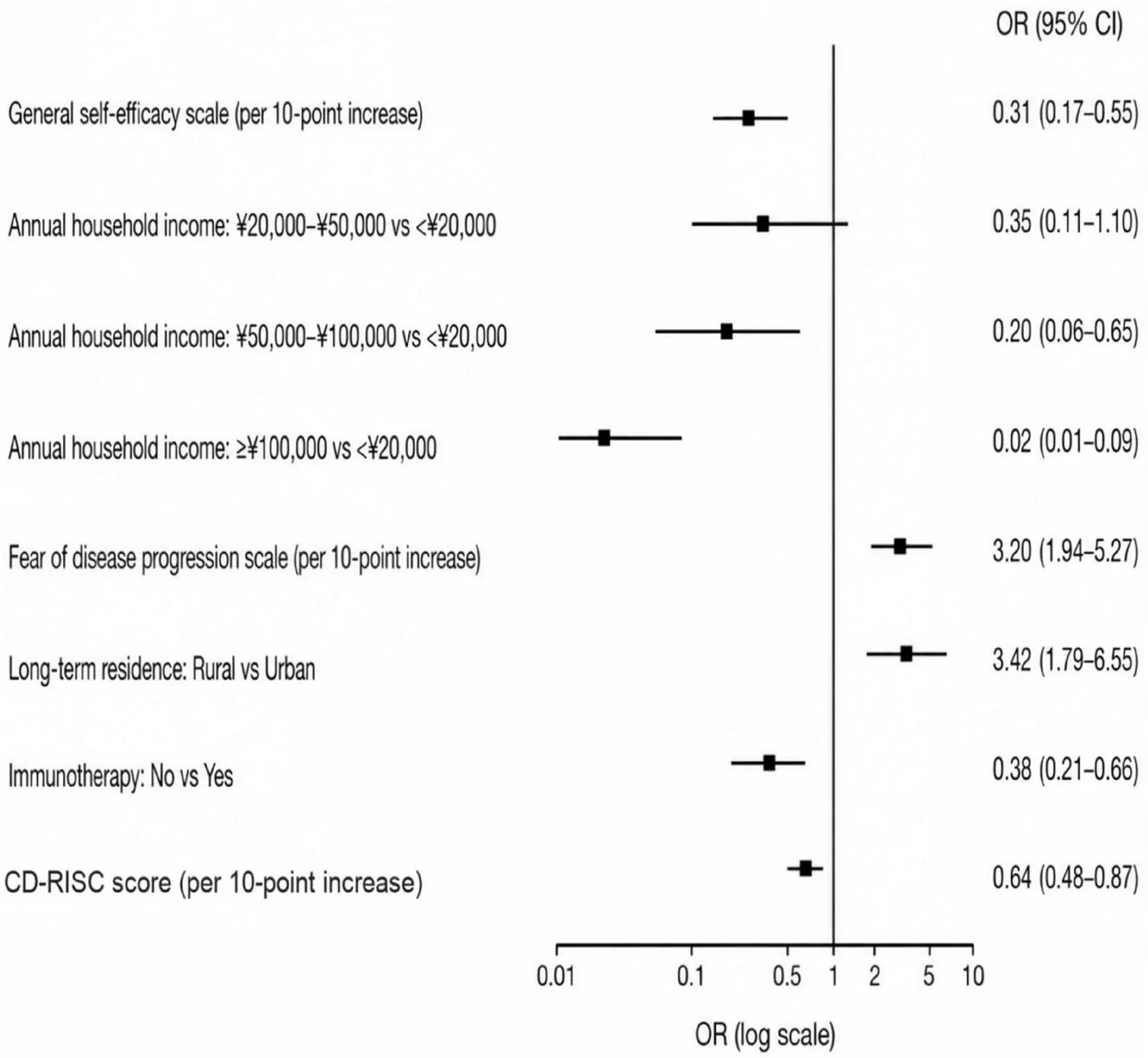
Forest plot of multivariable logistic regression analysis for predictors of financial toxicity at 6 months after discharge.

Candidate predictors were determined based on a comprehensive consideration of literature evidence, clinical relevance, availability in nursing assessment, temporal sequence of measurement, and results of univariate analysis. The final model was constructed taking into account the event number constraint and model parsimony. The final model included 8 free parameters, with 520 events of financial toxicity at 6 months after discharge, yielding an EPV of 65.0. Multivariable logistic regression analysis showed that GSES score, annual household income, FoP-Q-SF score, long-term place of residence, immunotherapy (yes/no), and CD-RISC score entered the final predictive model. The final model equation was: logit(P) = 4.605 − 1.174 × (GSES score/10) − 1.054 × I(annual household income 20,000–50,000 CNY) − 1.609 × I(annual household income 50,000–100,000 CNY) − 3.772 × I(annual household income ≥ 100,000 CNY) + 1.162 × (FoP-Q-SF score/10) + 1.229 × rural residence − 0.982 × no immunotherapy − 0.441 × (CD-RISC score/10); where P is the predicted probability of developing financial toxicity at 6 months after discharge; the reference categories are annual household income < 20,000 CNY, urban long-term residence, and receipt of immunotherapy. The multivariable logistic regression results are presented in Table 5 and visualized in Figure 1.

**TABLE 5:** Multivariable logistic regression analysis of predictors of financial toxicity at six months after discharge.

Variable	Category	β	SE	OR	95% CI	*P-*value
GSES (per 10 points)	20,000–50,000 CNY vs. < 20,000 CNY (reference)	−1.174	0.295	0.309	0.17–0.55	< 0.001
FoP-Q-SF (per 10 points)		1.162	0.255	3.197	1.94–5.27	< 0.001
CD-RISC (per 10 points)		−0.441	0.152	0.643	0.48–0.87	0.004
Annual household income		−1.054	0.586	0.348	0.11–1.10	0.072
Annual household income	50,000–100,000 CNY vs. < 20,000 CNY (reference)	−1.609	0.597	0.200	0.06–0.65	0.007
Annual household income	≥ 100,000 CNY vs. < 20,000 CNY (reference)	−3.772	0.675	0.023	0.01–0.09	< 0.001
Long-term place of residence	Rural vs. Urban (reference)	1.229	0.331	3.419	1.79–6.55	< 0.001
Immunotherapy	No vs. Yes (reference)	−0.982	0.286	0.375	0.21–0.66	< 0.001

### 3.4 Model discrimination and classification performance evaluation

**FIGURE 2:**
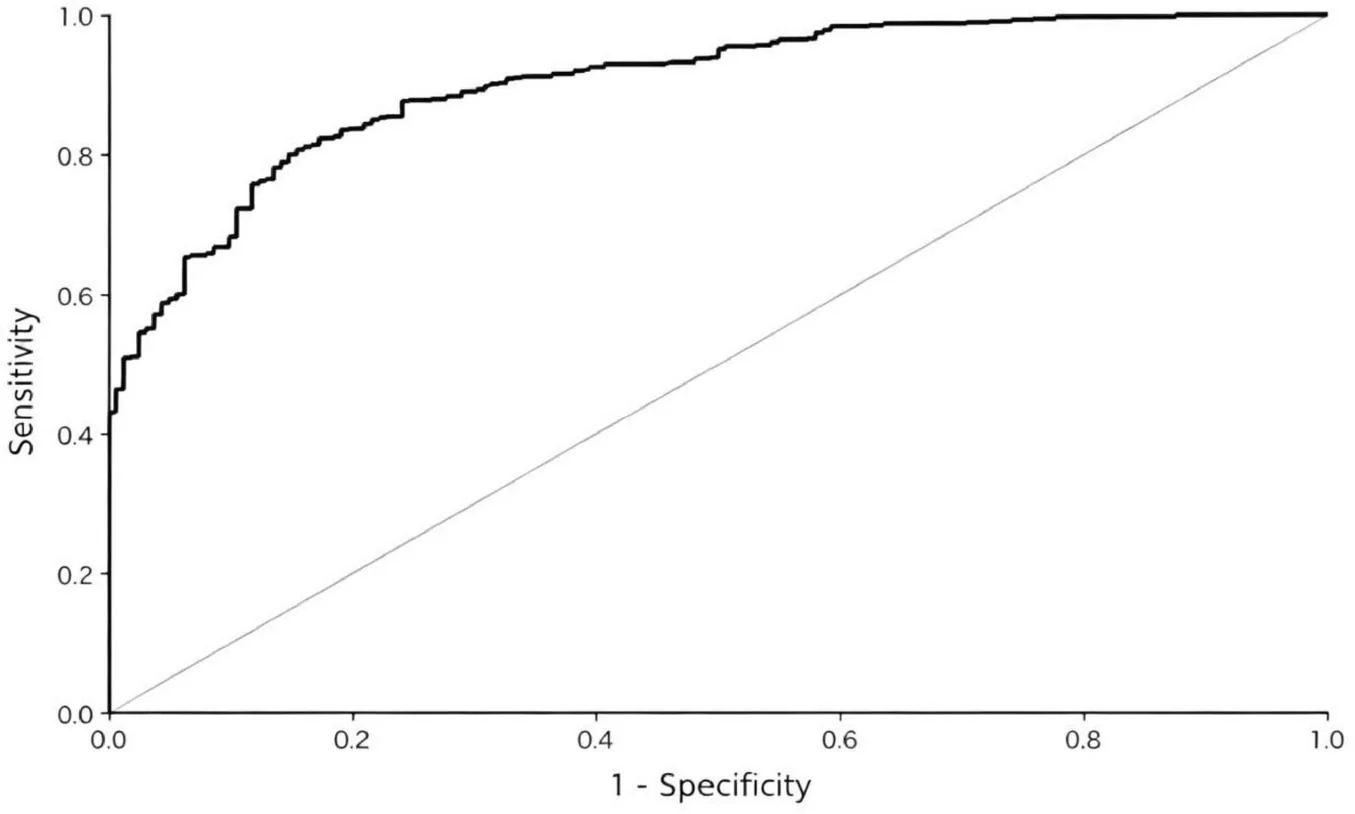
ROC curve of the prediction model for financial toxicity at 6 months after discharge.

The predicted probability of financial toxicity at 6 months after discharge was calculated for each patient based on the final multivariable logistic regression model. The average predicted risk of the model was 76.2%, which was close to the actual incidence of 76.2% in the sample. ROC curve analysis showed that the AUC of the model was 0.901, with a Bootstrap 95% CI of 0.883–0.927; after internal validation with 500 Bootstrap iterations, the corrected AUC was 0.894, indicating good discrimination. The ROC curve is shown in Figure 2. The optimal cutoff value determined by the Youden index was 0.757. At this cutoff, the sensitivity was 0.800, specificity was 0.852, positive predictive value was 0.945, negative predictive value was 0.570, and the Brier score was 0.104. The main performance indicators of the prediction model are summarized in Table 6.

**TABLE 6:** Main performance indicators of the prediction model for financial toxicity at six months after discharge.

Indicator	Result	Description
AUC	0.901	Bootstrap 95% CI: 0.883–0.927
Bootstrap-corrected AUC	0.894	Based on 500 Bootstrap internal validation iterations
Optimal cutoff value	0.757	Determined by Youden index
Sensitivity	0.800	–
Specificity	0.852	–
Positive predictive value	0.945	–
Negative predictive value	0.570	–
Brier score	0.104	Lower values indicate smaller overall prediction error

The calibration slope was 1.000, and the calibration intercept was –0.002, indicating nearly perfect calibration. After 500 bootstrap iterations with optimism correction, the optimism was –0.007 and the shrinkage factor was 1.007, suggesting minimal overfitting and excellent model stability.

**FIGURE 3:**
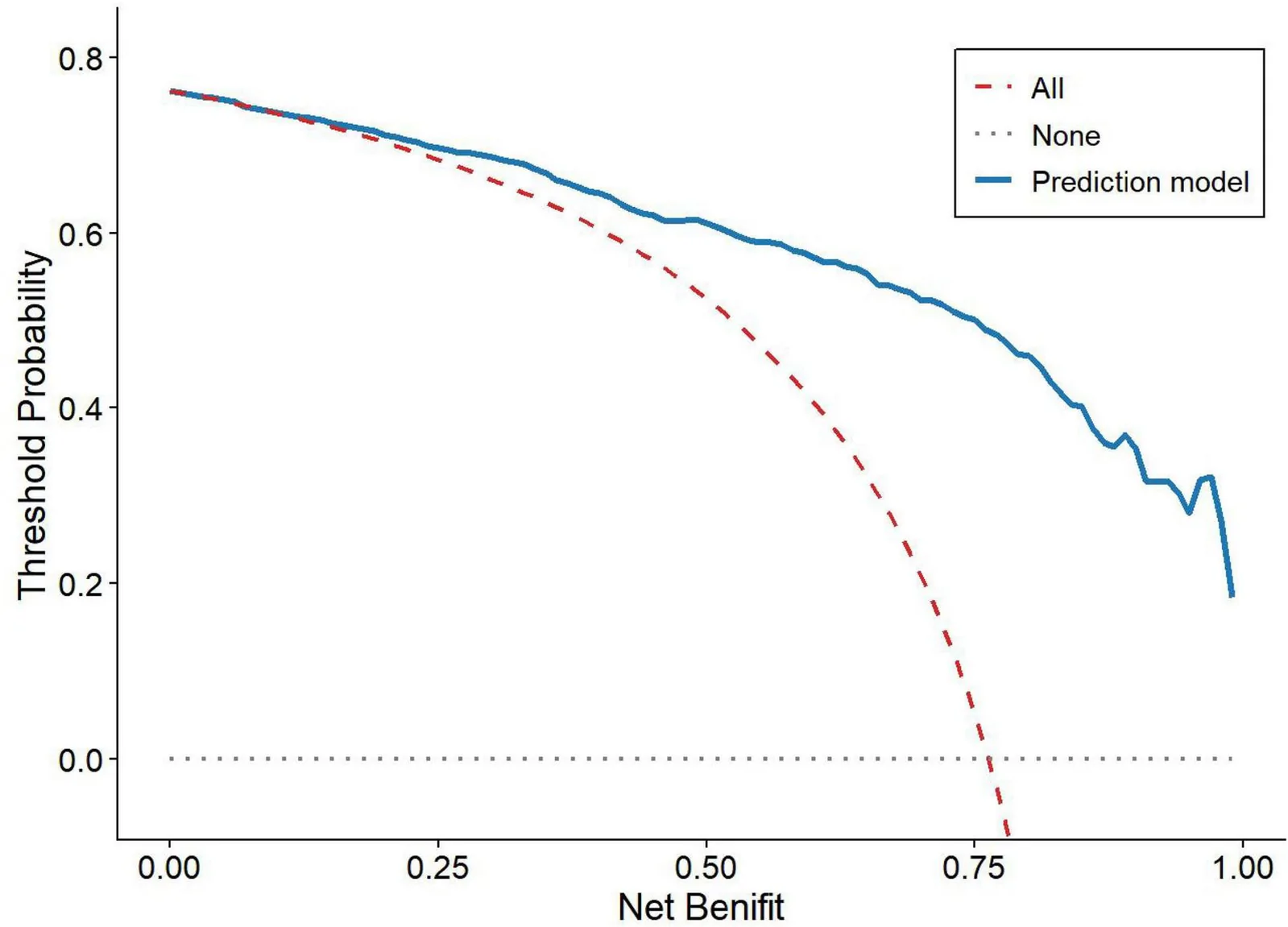
Decision Curve Analysis for the financial toxicity prediction model.

Decision Curve Analysis demonstrated that the prediction model provided a positive net benefit across threshold probabilities ranging from approximately 0.01 to 0.99, substantially exceeding both the “treat-all” and “treat-none” strategies (Figure 3). These findings support the clinical utility of the model for identifying high-risk patients and guiding stratified nursing interventions during transitional care.

### 3.5 Model calibration and Bootstrap internal validation

**FIGURE 4:**
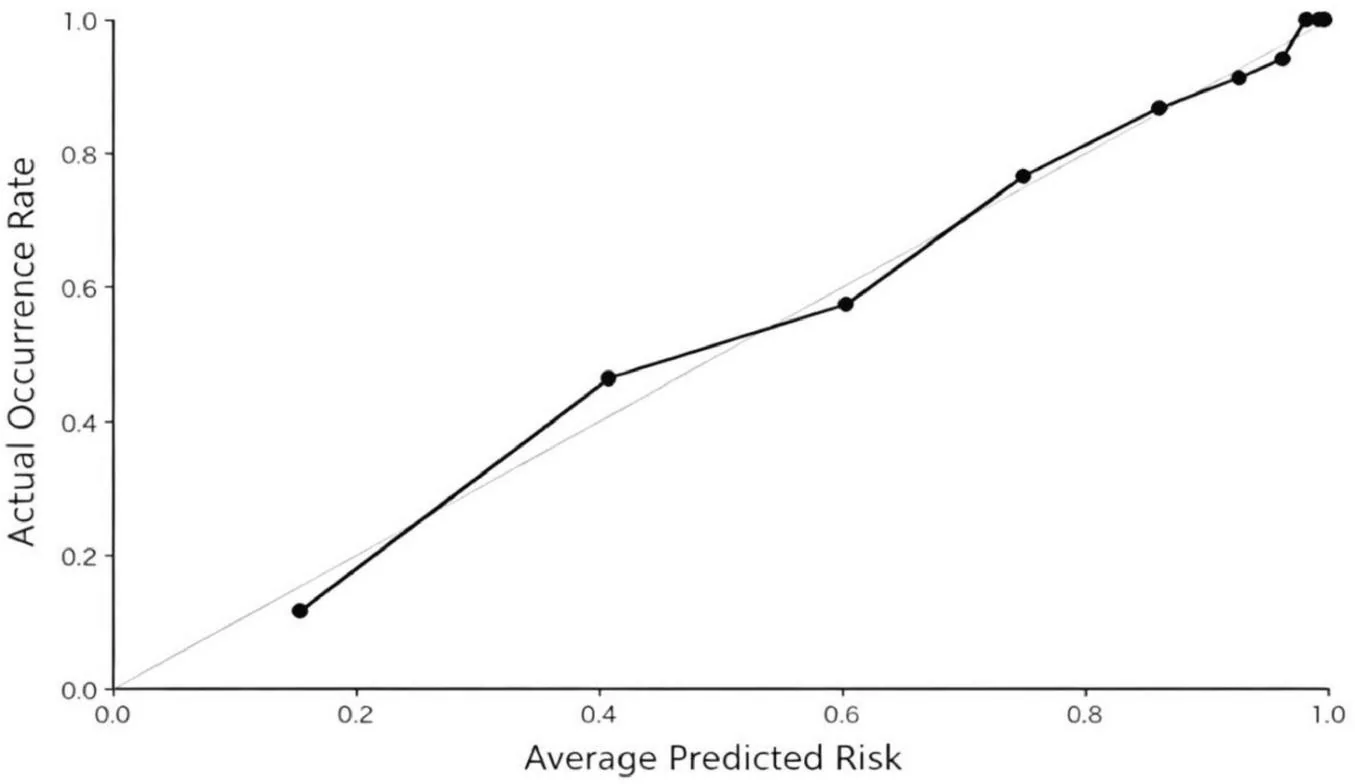
Calibration curve of the financial toxicity prediction model.

The Hosmer-Lemeshow goodness-of-fit test yielded χ^2^ = 5.00, *P* = 0.758, indicating no significant lack of fit. The calibration curve showed that the predicted risks were generally consistent with the observed incidence (Figure 4). After grouping by deciles of predicted probability, the observed incidence of financial toxicity was generally higher in higher predicted-risk groups than in lower predicted-risk groups. The predicted risks and observed incidences across the calibration groups are shown in Table 7. Patients were further divided into low-, intermediate-, and high-risk groups based on tertiles of predicted probability, dividing the cohort into three equal-sized groups. The low-risk group comprised 227 patients, with an incidence of financial toxicity of 41.0%; the intermediate-risk group comprised 228 patients, with an incidence of 88.2%; and the high-risk group comprised 227 patients, with an incidence of 99.6%. The observed financial toxicity and mean predicted risk across the three risk strata are shown in Table 8. The observed incidence of financial toxicity increased progressively across the three risk tiers, suggesting that the model has good risk stratification ability.

**TABLE 7:** Predicted risk and observed incidence by calibration groups.

Group	n	Mean predicted risk (%)	Observed incidence (%)
Decile 1	69	15.4	11.6
Decile 2	69	40.8	46.4
Decile 3	68	60.4	57.4
Decile 4	68	75.0	76.5
Decile 5	68	86.1	86.8
Decile 6	68	92.7	91.2
Decile 7	68	96.3	94.1
Decile 8	68	98.3	100.0
Decile 9	68	99.3	100.0
Decile 10	68	99.7	100.0

**TABLE 8:** Observed financial toxicity and mean predicted risk by risk stratum.

Risk stratum	n	Financial toxicity, n (%)	Mean predicted risk (%)
Low-risk	227	93 (41.0)	41.7
Intermediate-risk	228	201 (88.2)	88.1
High-risk	227	226 (99.6)	98.9

## 4 Discussion

### 4.1 High incidence of financial toxicity at 6 months post-discharge indicates prominent needs for transitional care among lung cancer patients

The incidence of financial toxicity was 76.2% among lung cancer patients at 6 months after discharge, indicating that the majority of patients still experienced substantial financial stress and economic distress after inpatient treatment during follow-up. Distinct from the cost burden merely incurred during hospitalization, financial toxicity at 6 months post-discharge reflects the overall burden brought by repeated re-examinations, medication, adjuvant therapy, maintenance systemic treatment, symptom management and household financial recovery. Although lung cancer treatment often involves prolonged and multimodal regimens, the active treatment phase for many patients typically spans several months, during which patients continue to incur substantial costs after discharge. After discharge, patients still need to cover costs for imaging tests, outpatient consultations, medications, radiotherapy, chemotherapy, targeted therapy and immunotherapy. They also face non-medical burdens such as transportation, caregiving, nutritional support and reduced household income. Consequently, financial toxicity does not subside upon hospital discharge, but tends to accumulate throughout follow-up and continuous treatment.

The incidence of financial toxicity observed in this study was consistent with the findings from national surveys on lung cancer patients in China. Liu et al. investigated 843 Chinese patients with lung cancer and reported that 77.0% suffered from financial toxicity of varying degrees, including 54.5% with mild and 22.5% with moderate to severe conditions (28). Using a COST-PROM total score ≤ 25 as the diagnostic cut-off for financial toxicity at 6 months post-discharge, the present study yielded an incidence of 76.2%. This indicates that financial stress remains highly prevalent even when patients transition from inpatient treatment to mid-term follow-up. Previous international studies have also reported substantial financial toxicity among patients with lung cancer and other malignancies; however, the reported prevalence varies across different healthcare systems, treatment settings, and assessment methods. Compared with countries with stronger financial protection mechanisms, patients in healthcare systems with higher out-of-pocket expenditures may experience greater financial distress. Differences in insurance reimbursement policies, accessibility of cancer care, household economic structures, and follow-up time points may partly explain the variation in prevalence across studies. Therefore, the high prevalence observed in the present study should be interpreted within the context of continuous treatment requirements and the socioeconomic characteristics of the study population. The similar results across the two studies suggest that financial toxicity among lung cancer patients is not a temporary issue for a small number of individuals under China’s medical payment and family care system, but a persistent burden experienced by a large proportion of patients throughout the entire treatment course.

Variations in the prevalence of financial toxicity across studies may be attributed to differences in assessment tools, cut-off values, healthcare payment policies, treatment phases, sample characteristics and follow-up time points. The 6-month post-discharge time point selected in this study carries distinct clinical implications. In a prospective cohort study of lung cancer patients, Friedes et al. assessed financial toxicity at diagnosis and the 6-month follow-up, and confirmed that its severity changes alongside treatment progression (11). It is worth noting that Friedes observed a decline in financial toxicity severity over time in their cohort, whereas our study found a persistently high prevalence at 6 months post-discharge. This discrepancy may reflect differences in healthcare settings, insurance coverage, the timing of assessment relative to the discharge period rather than diagnosis, and the specific characteristics of the study populations. The period of six months after discharge generally witnesses the transition from inpatient care to outpatient review, adjuvant therapy, maintenance systemic treatment and disease re-evaluation. During this phase, many patients continue to bear costs for treatment and follow-up, while their work capacity, income and household financial status may not have fully recovered. Accordingly, financial toxicity at 6 months post-discharge reflects both the lingering financial burden after hospitalization and patients’ financial adaptation in the mid-term follow-up stage.

Mechanistically, financial toxicity among discharged lung cancer patients arises from the combined effects of ongoing medical expenses, non-medical costs, income loss and subjective financial distress, rather than medical bills alone. Regular re-examinations, long-term medications, radiotherapy, chemotherapy, targeted therapy and immunotherapy lead to direct medical expenditures. Disease symptoms, treatment-related adverse reactions and physical decline hinder patients from returning to work and maintaining stable income. Increased caregiving from family members also results in indirect economic losses. For patients with low income, rural residence, complex treatment regimens or long-term treatment demands, medical insurance can only cover part of the expenses. Transportation, caregiving, nutritional support, work absence and out-of-pocket costs keep straining household disposable resources. Previous studies have demonstrated that financial toxicity is not only linked to affordability and out-of-pocket payments, but also associated with psychological distress, impaired quality of life and compromised treatment decisions (29, 30). Therefore, the high prevalence of financial toxicity observed in this study is clinically reasonable, which reflects the imbalance between sustained treatment needs and the recovery capacity of household finances. Although our prediction model did not include direct measures of non-medical costs or detailed insurance reimbursement data, these factors are partially reflected in the socioeconomic and residence variables included in the model. Future studies incorporating detailed medical expenditure and insurance claims data are warranted to further disentangle the contributions of these components.

The results of this study have direct implications for transitional care of lung cancer patients. Traditional discharge guidance mainly focuses on medication use, re-examination arrangements, symptom observation and complication identification, yet lacks systematic assessment of patients’ financial stress, understanding of medical insurance, subsequent payment capacity and social support resources. Persistent financial toxicity may impair patients’ follow-up adherence, treatment continuity, psychological status and quality of life (31). From a clinical transitional care perspective, the high prevalence observed in this study strongly mandates standardized early financial toxicity screening embedded in routine discharge workflows, paired with nurse-coordinated financial support and referral services. Unlike traditional discharge education limited to medication and follow-up reminders, nurses should proactively conduct structured financial burden assessment for all lung cancer inpatients before discharge: inquire about household disposable income, out-of-pocket treatment affordability, long-distance travel and caregiving non-medical expenses, and patients’ understanding of medical insurance reimbursement policies. For patients reporting obvious payment pressure, nurses can initiate structured financial assessment, provide basic financial guidance, and facilitate referral to professional financial counselors or social workers, including itemized breakdown of follow-up examination, maintenance drug and adjuvant therapy costs, step-by-step explanation of reimbursement rules, and referral channels for medical assistance and charitable resources. Furthermore, systematic financial risk assessment should be integrated into multi-disciplinary tumor care rounds, allowing nurses to flag high-risk patients to physicians, social workers and insurance counselors in advance for joint intervention planning. Assessment of financial burden should be incorporated into the transitional care system for lung cancer patients, with particular attention paid to those with long-term treatment requirements, income loss, heavy symptom burden, rural residence or inadequate family support. Early identification and stratified management allow medical staff to provide services including medical insurance policy explanation, expense consultation, social resource referral, psychological support and individualized follow-up plans, thereby transforming lung cancer nursing from simple disease management to comprehensive management covering physical symptoms, mental health and financial burden.

### 4.2 Psychosocial factors combined with clinical characteristics jointly affect the risk of financial toxicity

All patient-reported outcome variables were measured during hospitalization or before discharge, which precedes the assessment of financial toxicity at 6 months post-discharge. Therefore, these psychosocial factors serve as baseline predictors rather than concurrent correlates, supporting their temporal precedence in the prediction model.

Multivariate logistic regression analysis revealed that General Self-Efficacy Scale score, annual household income, Short Form of FoP-Q-SF score, long-term residence, immunotherapy administration and Connor-Davidson Resilience Scale score were independent predictors of financial toxicity at 6 months after discharge. Specifically, higher scores of general self-efficacy and psychological resilience, as well as higher annual household income, were associated with a lower risk of financial toxicity. In contrast, elevated fear of disease progression, rural residence and receipt of immunotherapy increased the risk of financial toxicity. These predictors collectively indicate that financial toxicity is a multidimensional outcome influenced by the interaction between socioeconomic vulnerability, treatment-related burden, and psychological adaptation. Socioeconomic factors, including household income and residential location, reflect patients’ objective financial capacity and accessibility to healthcare resources. Treatment-related factors, such as immunotherapy, represent cumulative medical expenditure and uncertainty regarding future treatment costs. Meanwhile, psychological factors, including self-efficacy, resilience and fear of disease progression, influence patients’ ability to cope with economic stress and their subjective perception of financial burden. Therefore, financial toxicity should not be regarded as a simple consequence of medical expenses, but rather as the result of complex interactions among economic resources, healthcare demands and psychological coping capacity. These findings indicate that financial toxicity among lung cancer patients at 6 months post-discharge is not merely attributable to medical expenses, but results from the combined effects of psychosocial resources, household financial capacity, treatment regimens and accessibility to medical resources.

This study also found that increased general self-efficacy was correlated with reduced risk of financial toxicity. Self-efficacy reflects patients’ confidence in coping with illness, solving problems and adhering to health-related behaviors. Patients with higher self-efficacy tend to actively acquire treatment-related information, understand medical insurance and reimbursement policies, communicate with medical staff about cost concerns, and adopt positive problem-solving strategies during follow-up visits, medication administration and lifestyle adjustments. Previous studies have demonstrated that self-efficacy exerts a mediating effect between socioeconomic status and financial toxicity, and a higher level of self-efficacy can enhance patients’ sense of control over resource utilization and disease management (32). Accordingly, self-efficacy cannot directly cut medical costs, but may mitigate the adverse impacts of financial strain on subjective distress and treatment decision-making by improving patients’ abilities to obtain information, communicate and resolve practical problems.

Higher psychological resilience was also associated with a decreased risk of financial toxicity. Patients with greater resilience tend to possess stronger emotional regulation, stress tolerance and adaptive capacity when confronting cancer diagnosis, treatment-related adverse reactions and household financial strain. After discharge, lung cancer patients still face regular re-examinations, uncertainty regarding treatment outcomes and changes in family roles, with financial pressure often coexisting with disease-related stress. Those with low resilience are more likely to perceive financial burden as a state of loss of control, helplessness and long-term threat, which exacerbates the subjective experience of financial toxicity. In contrast, individuals with high resilience are better able to mobilize family support, social resources and positive coping strategies. Previous studies on financial toxicity and psychosocial resources among cancer patients have indicated that insufficient psychological resources may amplify the detrimental effects of financial burden on quality of life and emotional status (33–35). Therefore, psychological resilience serves as a critical protective factor against financial toxicity in lung cancer patients.

Elevated score on the FoP-Q-SF was correlated with an increased risk of financial toxicity. Fear of disease progression reflects patients’ concerns about cancer recurrence, metastasis, treatment failure and uncertainties about the future (36–38). During the long-term treatment and follow-up of lung cancer, uncertainties regarding disease status are often compounded by concerns over medical costs. Patients with intense fear of disease progression tend to experience persistent worries about subsequent examinations, treatment escalation, medication expenses, family caregiving and income disruption. Particularly with the continuous advancement of targeted therapy, immunotherapy and multi-line treatment regimens, patients’ anxieties over the necessity of ongoing treatment, affordability of subsequent interventions and family arrangements in the event of disease progression can directly translate into perceived financial strain. Accordingly, fear of disease progression is not only an indicator of psychological distress, but also an important psychological warning sign for the future risk of financial toxicity.

Annual household income emerged as a key socioeconomic predictor in the model. Compared with patients whose annual household income was less than 20,000 RMB, those with an income ranging from 50,000 to 100,000 RMB and no less than 100,000 RMB had a markedly lower risk of financial toxicity, and the reduction was most prominent in the group with income ≥ 100,000 RMB. This finding aligns with the essence of financial toxicity, which depends not on the absolute amount of medical expenses, but on the impact of medical spending relative to household affordability. For low-income families, identical expenditures on examinations, treatment, transportation, caregiving and nutritional support are more likely to lead to tight cash flow, cutbacks on daily spending, borrowing, and compromised treatment decisions. Accumulated evidence has confirmed that low income, limited affordability and socioeconomic disadvantage are major risk factors for financial toxicity among cancer patients (39–41). Hence, annual household income acts not merely as a demographic variable, but also as a critical indicator for evaluating long-term financial capacity and conducting risk stratification in lung cancer patients.

In terms of long-term residence, rural residents presented a higher risk of financial toxicity at 6 months post-discharge than urban counterparts. This result can be well explained by practical circumstances. Rural patients usually incur higher costs on transportation, accommodation and accompanying care when seeking treatment at specialized hospitals. Additionally, they tend to have less stable income, less convenient medical insurance reimbursement, poorer access to healthcare resources and insufficient support for disease management compared with urban patients. Even if direct medical costs are partially covered by medical insurance, recurring non-medical expenses arising from repeated follow-up visits, examinations, travel and caregiving continue to impose a heavy burden on families. Previous studies have also demonstrated that residential location, healthcare accessibility and socioeconomic disparities affect the severity of financial toxicity in cancer patients (42, 43). Accordingly, rural residence represents comprehensive gaps in medical accessibility, financial affordability, caregiving resources and information acquisition, rather than a mere geographical difference.

In terms of treatment-related factors, this study found that patients who did not receive immunotherapy had a lower risk of financial toxicity than those who did, indicating that lung cancer patients undergoing immunotherapy are more vulnerable to this issue. Immunotherapy is generally characterized by long treatment cycles, high costs, frequent efficacy assessments and ongoing management of adverse reactions. Even though some immunotherapeutic drugs are covered by medical insurance, patients still need to bear out-of-pocket expenses for medications, examinations and transportation, as well as substantial time costs. Furthermore, the persistent and unpredictable nature of expenses related to immunotherapy tends to intensify patients’ concerns about future affordability. For patients requiring long-term maintenance therapy or combination regimens, financial toxicity is not merely a short-term pressure caused by one-off inpatient costs, but a cumulative financial burden throughout the entire treatment course. Therefore, patients receiving immunotherapy should be prioritized for post-discharge screening of financial toxicity and targeted financial support.

The present findings reveal that financial toxicity among lung cancer patients at 6 months after discharge results from the combined effects of psychosocial resources, household financial status, residential environment and treatment regimens. General self-efficacy and psychological resilience reflect patients’ internal resources to cope with illness and financial stress. Fear of disease progression indicates the psychological burden stemming from uncertainties regarding subsequent treatment and disease prognosis. Household income and residential location mirror patients’ financial affordability and accessibility to medical resources, while immunotherapy represents sustained treatment costs and payment-related uncertainties. For nursing practice, assessment of financial toxicity should go beyond simply asking whether patients can afford medical expenses. It is necessary to simultaneously evaluate patients’ psychological coping capacity, disease-related worries, household income, residential location, treatment plans and follow-up requirements. Comprehensive assessment of all these factors enables accurate identification of patients at high risk of financial toxicity at 6 months post-discharge, and provides a solid basis for stratified follow-up and individualized supportive interventions. From a nursing perspective, these findings suggest that assessment of financial toxicity should extend beyond direct economic indicators. Patients’ psychological resources, treatment expectations, disease-related concerns and healthcare accessibility should also be incorporated into routine risk assessment. Such a comprehensive approach may help nurses identify patients whose financial distress is likely to emerge during the post-discharge period and provide opportunities for early supportive intervention.

### 4.3 Screening and stratified nursing management of financial toxicity based on risk prediction model

This study established a risk prediction model for financial toxicity at 6 months after discharge among lung cancer patients based on multivariate logistic regression. The model incorporated general self-efficacy, annual household income, fear of disease progression, long-term residence, immunotherapy and psychological resilience as predictive variables, with an AUC of 0.901 and a Bootstrap-corrected AUC of 0.894 after rigorous 500-time Bootstrap resampling internal validation, indicating satisfactory discriminative ability. Compared with simple analysis of the incidence and associated factors of financial toxicity, this prediction model integrates multi-dimensional indicators to calculate individual risk probabilities, enabling nurses to identify high-risk patients before discharge or at the early stage of follow-up. The novelty of this model is reflected in several aspects. First, this study focused on financial toxicity at 6 months after discharge, a clinically important transition period when patients continue outpatient treatment, follow-up examinations and long-term disease management. Second, unlike previous studies that mainly emphasized demographic and clinical characteristics, the present model incorporated patient-reported psychological factors, including self-efficacy, resilience and fear of disease progression, providing a more comprehensive perspective on financial toxicity risk. Third, all predictors included in the model can be obtained during hospitalization or before discharge, which enhances its feasibility for nursing-led screening and transitional care. Since all variables included in the model can be obtained through routine inpatient assessment and standard nursing work, the model has low application barriers and is feasible to be embedded into hospital electronic medical record systems as a concise calculation module for routine use in discharge planning and transitional care procedures (44), supporting universal early financial toxicity screening across all lung cancer inpatients before discharge. In clinical practice, nurses can collect required data covering socioeconomic characteristics, treatment-related factors and psychological assessment results before discharge to calculate individual predicted risk of financial toxicity. Moderate- and high-risk patients can be provided with targeted interventions including financial counseling, medical insurance guidance, social resource referral, psychological support and intensified follow-up. This working mode enables proactive identification and individualized management of financial toxicity, instead of merely depending on patients’ spontaneous reporting of economic hardship.

In terms of classification performance, the model yielded a sensitivity of 0.800, a specificity of 0.852, a positive predictive value of 0.945 and a negative predictive value of 0.570 at the optimal cut-off value of 0.757. These results demonstrate that the model performs well in identifying patients at high risk of financial toxicity. Patients classified as high-risk are highly likely to develop financial toxicity during follow-up, thus requiring priority assessment and targeted interventions delivered through standardized nurse-led financial counseling. Given the high overall incidence of financial toxicity at 6 months post-discharge in this cohort, the negative predictive value was relatively low, indicating that a low-risk prediction cannot completely rule out the subsequent occurrence of financial toxicity. Accordingly, this model is not intended as a diagnostic tool for financial toxicity, nor can it replace routine assessment of financial burden. Instead, it is suitable for risk stratification on the basis of universal screening, assisting clinical teams in determining follow-up intensity, resource allocation and intervention priorities.

This study also revealed that the actual incidence of financial toxicity was 41.0, 88.2, and 99.6% in the low-, medium- and high-risk groups, respectively, showing a distinct increasing trend and verifying the favorable risk stratification capacity of the model. Notably, over 40% of patients in the low-risk group still experienced financial toxicity, which suggests that the so-called low risk in this study is relative rather than absolute. This finding carries important implications for nursing practice. Universal screening for financial toxicity is recommended among all lung cancer patients, who should receive routine financial burden assessment and relevant information on medical expense policies during basic discharge guidance. Meanwhile, the prediction model can help nurses identify patients at medium and high risk, so as to deliver more intensive and proactive follow-up alongside personalized financial counseling services covering breakdowns of follow-up medication and examination costs, detailed interpretation of medical insurance reimbursement rules, and referrals to hospital social workers for charitable medical aid resources. For patients with relatively low predicted risk, nursing staff only need to provide standardized group financial guidance and arrange regular COST-PROM reassessment at mid-term follow-up to dynamically monitor changes in financial pressure, without deploying intensive one-on-one consultation resources. For patients falling into the medium-risk stratum, nurses should conduct supplementary semi-structured interviews to quantify monthly out-of-pocket costs and pinpoint major economic burdens such as long-distance transportation and caregiver income loss, while carrying out brief psychological intervention targeting elevated fear of disease progression to ease patients’ anxiety about unpredictable future treatment expenses, and appropriately shorten the interval of routine follow-up visits to strengthen real-time monitoring of financial distress. Patients identified as high-risk by the model demand comprehensive, multidisciplinary collaborative nurse-led transitional care interventions: nurses first complete a full inventory of household economic burdens including medical debt and indirect caregiving expenses, then coordinate face-to-face communication with hospital insurance specialists to sort out reimbursable and self-funded treatment items and assist with applications for serious illness subsidies; nurses also deliver targeted psychosocial support to boost patients’ resilience and self-efficacy via peer experience-sharing and regular one-on-one counseling, and act as case managers to link rural patients with community medical services to cut repeated travel expenditures, arranging monthly telephone or home follow-up throughout the 6-month post-discharge window to adjust supportive measures promptly once financial strain worsens.

From the perspective of nursing management, financial toxicity screening should be embedded into the discharge planning and transitional care procedures for lung cancer patients. Previous expert consensus has emphasized that screening and management of financial toxicity should serve as an essential component of oncology care, and medical teams should proactively identify patients’ economic distress and financial risks rather than passively waiting for patients to raise financial concerns (7). This perspective is consistent with the application scenario of the model established in this study. Lung cancer patients require continuous outpatient revisits, examinations and treatments within 6 months after discharge, and financial burden is closely intertwined with symptom management, psychological adaptation, treatment adherence and family care. Our findings support embedding FT screening into discharge planning because the model enables nurses to identify high-risk patients prior to discharge and initiate targeted interventions during the transitional period, thereby shifting from reactive to proactive management of financial burden.

The present model further suggests that nursing interventions for financial toxicity should not be simplified to mere cost interpretation, but expanded to comprehensive supportive interventions. For patients identified as high-risk, targeted nursing interventions should cover four core dimensions. First, medical staff should elaborate on treatment-related expenses, medical insurance reimbursement procedures, and the composition of subsequent follow-up costs to alleviate patients’ anxiety and decision-making delays caused by information asymmetry through tailored financial counseling. Second, systematic assessment should be performed on patients’ non-medical burdens, including transportation expenditures, caregiving costs, nutritional support expenses, work absence and income disruption. Third, close attention should be paid to the amplifying effects of fear of disease progression, severe symptom burden and poor psychological resilience on patients’ perceived financial stress, with psychological support integrated into routine nurse-led follow-up. Fourth, standardized follow-up reminder and resource referral mechanisms should be established to assist patients in accessing professional resources including medical insurance consultation, charitable assistance, social work services, psychological support and community nursing care. Only by integrating financial guidance, psychological intervention, symptom management and social support can the management of financial toxicity be truly incorporated into the whole-course nursing system for lung cancer patients. The recommendation for nurse-led financial counseling is supported by emerging evidence. A survey of 535 oncology nurses showed that 76% believed they should actively address financial toxicity, yet 87% lacked formal training (45). Training programs such as FINassist-Train have been shown to improve nurses’ comfort and referral practices (46). Given their established therapeutic relationships and continuity of care, nurses are well-positioned for early screening and referral to specialized financial navigators or social workers (46). We therefore conceptualize this role as early screening, detection, and referral within a multidisciplinary team, rather than independent delivery of complex financial advice.

The risk prediction model established in this study demonstrated promising discriminative ability and risk stratification performance. Rather than replacing the COST-PROM scale or comprehensive clinical judgment by nurses, this model may serve as a potential auxiliary tool to assist clinicians and nurses in identifying patients at high risk of financial toxicity. However, external validation in diverse populations and healthcare settings is required before its routine clinical implementation. Given the high prevalence of financial toxicity among lung cancer patients, future nursing practice should integrate universal screening with risk stratification. Basic financial burden assessment should be implemented for all lung cancer patients, while more proactive, intensive and comprehensive transitional care interventions led by frontline nurses, paired with customized financial counseling, should be delivered to individuals categorized as moderate-to-high risk by the model. Such strategies can promote the standardized management of financial toxicity by shifting traditional empirical judgment toward structured screening and stratified nursing care.

## 5 Limitations of the study

This study has several limitations. First, this is a single-center prospective cohort study, and all participants were recruited from a single hospital. The disease profiles, treatment patterns, medical insurance payment conditions and follow-up management strategies of the included patients may present institutional specificity. Although internal validation was performed using the Bootstrap method, the established model requires further external validation in populations from different regions, hospitals at diverse levels and varied medical payment settings. Although consecutive enrollment was applied, selection bias cannot be completely excluded because this study was conducted in a single institution and included patients who were able to complete baseline assessments and follow-up evaluations. Therefore, the characteristics of the included participants may differ from those of patients in other clinical settings, which may limit the generalizability of the model.

Second, the primary outcome of this study was financial toxicity at 6 months after discharge, which reflects the mid-term economic burden of lung cancer patients but cannot fully illustrate the dynamic changes of financial toxicity throughout the entire course of diagnosis, hospitalization, early post-discharge stage and long-term treatment. Future studies should set multiple follow-up time points to explore the longitudinal trajectory and persistent risk of financial toxicity.

Third, the prediction model was constructed based on demographic, socioeconomic, clinical and patient-reported outcome variables, while objective economic indicators, such as out-of-pocket expenses, medical insurance reimbursement rate, drug costs, transportation and caregiving expenditures, and dynamic income changes, were not included. We acknowledge that predictors such as household income and employment status may change over time, and the use of baseline measurements may not fully capture these dynamics. Additionally, baseline financial toxicity was not assessed in this study, as our primary focus was on predicting incident financial toxicity at 6 months post-discharge using pre-discharge predictors; baseline FT could serve as a strong predictor of subsequent FT, but its exclusion was intentional to enable risk identification before discharge without requiring a pre-existing FT assessment. Furthermore, family size was not captured in the model, although it may influence household financial capacity. Further studies incorporating medical insurance settlement data and detailed household economic information, including household composition, are warranted to improve the explanatory power and predictive performance of the model. In addition, several predictors included in the model were derived from patient-reported questionnaires, including psychological resilience, fear of progression and self-efficacy. Although validated instruments were used, self-reported measures may still be affected by subjective interpretation, emotional status and response bias. Furthermore, residual confounding may exist because some potential factors, such as detailed household financial conditions, social support, employment changes and reimbursement characteristics, were not fully captured in this study.

Fourth, despite the favorable discriminative ability of the model, the results were derived from single-center samples and internal validation, which may lead to potential optimism bias. Owing to the relatively high incidence of financial toxicity in the current cohort, the positive and negative predictive values may vary in other populations. Therefore, external validation and clinical application studies are required to clarify the practical value of this model in real nursing scenarios.

Finally, although internal validation using bootstrap resampling with optimism correction and shrinkage adjustment was performed to reduce overfitting, the model was developed and validated within a single-center cohort and has not yet undergone external validation in independent populations. We explicitly acknowledge this limitation and plan to conduct external validation studies in multi-center settings in future research.

## Data Availability

The raw data supporting the conclusions of this article will be made available by the authors, without undue reservation.
